# APRISMA-compliant systematic review and meta-analysis determining the association of miRNA polymorphisms and risk of congenital heart disease

**DOI:** 10.1097/MD.0000000000017653

**Published:** 2019-11-11

**Authors:** Xing-Yan Li, Kun Chen, Zheng-Tao Lv

**Affiliations:** a Department of Orthopedics, The Third Affiliated Hospital of Guangxi Medical University, Nanning, Guangxi; b Department of Orthopedics, The First Affiliated Hospital of USTC, Division of Life Sciences and Medicine, University of Science and Technology of China, Hefei, Anhui; c Department of Orthopedics, Tongji Hospital, Tongji Medical College, Huazhong University of Science and Technology, Wuhan, China.

**Keywords:** congenital heart disease, meta-analysis, microRNA, polymorphism, systematic review

## Abstract

**Purpose:**

Recent genetic association studies showed conflicting results on the relationship of miRNA single-nucleotide polymorphisms (SNPs) and congenital heart disease (CHD) risk. The purpose of the present systematic review was to collect the current available evidences to evaluate the association between miRNA polymorphisms and CHD risk.

**Methods:**

Four electronic databases including PubMed, EMBASE, ISI Web of Science, and CENTRAL were extensively searched for relevant studies published before February, 2019. Observational studies determining the association between miRNA polymorphisms and risk of CHD were included. Risk of bias was evaluated using the Newcastle-Ottawa Scale by 2 independent researchers. Major characteristics of each study and estimation of effect size of individual locus polymorphism were summarized. In addition, meta-analysis was performed to quantify the associations between miRNA polymorphisms and CHD risk.

**Results:**

Nine studies containing 6502 CHD patients and 6969 healthy controls were included in this systematic review. Ten loci in 9 miRNAs were reported. Only rs11614913 in miR-196a2 was determined to have significant associations with CHD susceptibility, which was supported by meta-analysis (CC vs CT+TT: odds ratio 1.54, 95% confidence interval 1.30, 1.82; *P* < .00001). A strong evidence indicated lack of association between rs2910164 in miR-146a and CHD. Limited or conflicting evidences were found for the associations of the other variants (rs11134527, rs139365823, rs76987351, rs3746444, rs4938723, rs2292832, rs41291957, rs895819) and risk of CHD.

**Conclusions:**

Locus polymorphisms in miRNAs are not generally associated with CHD. Only rs11614913 was found to have significant associations with CHD. Further studies will be needed, using larger populations of different ethnicities, to obtain a better understanding of these associations.

## 1 Introduction

Congenital heart disease (CHD), accounting for higher instances of infant morbidity than any other kind of malformation globally, is the most common congenital defect in newborn babies. This disorder represents several heart anomalies, including valve defects, septal defects, and outflow tract anomaly. CHD is present since birth. Prevalence of CHD at birth was 9.3, 8.2, and 6.9 per 1000 surviving births among Asian, European, and North American populations, respectively.^[1]^ Although advances in surgical techniques and nursing procedures have significantly improved the survival rate, the mortality due to CHD remains high.^[2]^ CHD is associated with emotional, physiological, and socioeconomic factors not only for CHD patient, families, but also the whole society.^[3]^ Therefore, investigation of the cause of CHD is needed.

It has long been considered that the onset of CHD is caused by both genetic and environmental factors. Epidemiological studies have revealed that majority of CHDs occur genetically, whereas environmental factors play a less important role in the pathogenesis of CHD. Strong familial accumulation of CHD in first-degree relatives was observed.^[4]^ Chromosome number variations (aneuploidy),^[5]^ copy number variations,^[6,7]^ and inherited point mutations^[8,9]^ are well-known identified genetic causes of CHD. Several genes have been reported to contribute to the genesis of CHD, including TBX1, NKX2.5, and TBX5.^[10]^

Recent studies have shown that microRNA (miRNA) genes play crucial role in the inheritance of CHD.^[11]^ MiRNAs are a family of single-stranded noncoding RNAs (∼22 nucleotides) that regulate gene expression by interfering with the expression of target messenger RNAs (mRNAs).^[12]^ MiRNAs are first transcribed as pri-miRNAs, which are further processed into pre-miRNAs by RNase III-type enzyme, Drosha. Pre-miRNAs are hereafter transported to the cytoplasm and cleaved into mature miRNAs by ribonuclease, Dicer.^[13]^ MiRNAs are involved in a large number of developmental and physiological processes. MiRNAs play crucial roles in controlling diverse aspects of cardiac function, such as cardiocyte growth, contractility, integrity of the ventricular wall, gene expression, and maintenance of normal rhythm.^[14]^ The general significance of miRNA for cardiovascular system was confirmed by cardio-myocyte-specific deletions of Dicer^[15]^ and Dgcr8,^[16]^ 2 important enzymes involved in miRNA maturation machinery. CHD-associated miRNAs are usually expressed in tissue-specific and developmental stage-specific manner.^[17]^ For instance, miR-34b/c and miR-499 are specifically expressed in the heart,^[18]^ whereas miR-146a is mainly expressed in vascular smooth muscle cell.^[19–21]^

Genetic association studies hold great prospect for the classification of diseases with genetic basis. Single-nucleotide polymorphism (SNP) refers to single-nucleotide variations in a genetic sequence among people. SNP has been widely used in genetic association studies recently. Since Xu et al^[22]^ initially reported the association of miRNA gene polymorphisms and CHD, other researchers^[23–30]^ attempted to validate the association and to discover new loci. The outcomes were inconsistent.

A systematic review has not been conducted to determine the association between miRNA gene polymorphisms and risk of CHD. Here, we propose to collect the current body of evidence to identify those miRNA SNPs that could alter the risk of CHD.

## 2 Materials and methods

This systematic review was reported in accordance with the Preferred Reporting Items for Systematic Review and Meta-Analyses (PRISMA) guidelines.^[31]^

### 2.1 Literature search strategy

The computerized literature search was systematically conducted using four online electronic databases, including PubMed, EMBASE, ISI Web of Science, and CENTRAL. All the relevant literature was published before February, 2019. A combination of Medical Subject Headings (MeSH) together with free terms was utilized to locate all the potentially eligible publications without any language restrictions. We used the following search string in PubMed: (Single Nucleotide Polymorphism or polymorphism or SNP or SNPs or “Polymorphism, Single Nucleotide”[Mesh]), (“MicroRNAs”[Mesh] or microRNAs or miRNAs or micro RNA or pre-miRNA or pre-microRNA) and (CHD or congenital heart defect). In addition, the bibliographic lists of relevant reviews and full-text articles were manually examined for eligible studies. This process was accomplished independently by 2 investigators.

### 2.2 Inclusion and exclusion criteria

Studies satisfying the following criteria were included for review: published studies on the relationship between miRNA gene polymorphisms and CHD susceptibility; observational studies (case-control or cohort studies) on humans; CHD diagnosed on the basis of clinical examination and confirmed during the surgeries; (4) studies that were required to provide available data to calculate the odds ratio (OR) and the corresponding 95% confidence interval (95% CI).

Correspondingly, studies were excluded if they met with the following criteria: reviews, case reports, conference abstracts, and editorials; data that overlapped with previous publications. If potentially eligible studies reported overlapped data, the most comprehensive one was included in our systematic review.

### 2.3 Methodological quality assessment

The methodological quality of eligible studies was evaluated following the Newcastle-Ottawa Scale (NOS) for observational studies.^[32]^ A “star system" was applied to judge each study on 3 broad perspectives: selection of the case and control groups; comparability of the case and control groups; and ascertainment of either the exposure or outcome of interest for case–control studies. Studies scored ≥7 stars were deemed as low risk of bias, and those scored <4 stars were deemed as high risk of bias, studies were labeled with unclear risk of bias if they scored 4, 5, or 6 stars. Disagreements between investigators were settled by discussion until consensus was reached.

### 2.4 Data extraction

In compliance with the predefined criteria, following information was meticulously extracted independently by 2 reviewers from all qualified articles: surname of the primary author; year of publication; country where the study was performed; study design; numbers of CHD case and control subjects; miRNA genes and corresponding variants; *P* value for Hardy–Weinberg equilibrium (HWE); genetic model of inheritance; crude ORs for the associations between miRNA polymorphisms and risk of CHD along with the corresponding *P* values. Once encountering discrepancy during this process, 2 authors reinspected the article together and reached an agreement by mutual discussion.

### 2.5 Statistical analysis

The disappearance of HWE was examined in the control group of each study using the *χ*^2^ goodness-of-fit test. The correlations between miRNA polymorphisms and CHD risk were measured using crude OR accompanied by 95% CI. We used “best-evidence” synthesis method to evaluate the association between miRNA polymorphisms and CHD risk.^[33,34]^ Evidence was defined as “generally consistent" if ≥75% of the included studies/cohorts reported consistent results. The level of evidence was defined according to following criteria: strong evidence: ≥2 studies with low risk of bias and generally consistent findings in all included studies/cohorts; moderate evidence: one study with low risk of bias, ≥2 studies with high risk of bias and generally consistent findings in all included studies/cohorts; limited evidence: 1 study with low risk of bias or >2 studies with high risk of bias, and generally consistent findings in all included studies/cohorts; insufficient evidence: a finding in one study with high risk of bias. If <75% studies/cohorts reported consistent findings, then that evidence was defined as conflicting evidence.

In addition to the “best-evidence” synthesis method, we employed meta-analysis approach for a better understanding of the association between miRNA polymorphisms and CHD risk. We did not make any assumption about the genetic model of inherence, in advance. The best-fitting genetic model was confirmed by a model-free approach to avoid an inflated false positive error.^[35]^ If A variant was the polymorphism of interest that could potentially alter the CHD risk, then OR1, OR2, and OR3 were calculated for genotypes AA vs aa, Aa vs aa, and AA vs Aa for each polymorphism to capture the magnitude of genetic effect and to identify the most appropriate genetic model. The most plausible genetic model used for meta-analysis was confirmed according to the relationships between the 3 pairwise comparisons as follows:

1. Recessive model: if OR1 = OR3≠1 and OR2 = 1
2. Dominant model: if OR1 = OR2≠1 and OR3 = 1
3. Complete over-dominant model: if OR1 = 1, OR2 = 1/OR3≠1
4. Co-dominant model: if OR1>OR2>1 and OR1>OR3>1, or OR1<OR2<1 and OR1<OR3<1.

Meta-analysis was performed using the genetic model that was confirmed by the model-free approach. The between-study heterogeneity was measured using *Q* statistical test and *I*^2^ test.^[36]^ Random-effect or fixed-effect models were selected for statistical analysis with the presence (*P* < .1, *I*^2^ >50%) or absence of heterogeneity (*P* > .1, *I*^2^ < 50% indicates acceptable heterogeneity), respectively.^[37]^ The Leave-one-out sensitivity analysis was conducted to test the robustness of association between miRNA polymorphism and CHD risk. Egger regression and Begg rank correlation tests were performed to assess the publication bias (Stata version 12.0, StataCorp LLC, College Station, TX).^[38]^ Forest plots were generated using RevMan 5.3 software (Copenhagen: The Nordic Cochrane Center, The Cochrane Collaboration, 2014).

## 3 Results

### 3.1 Literature search

**Figure 1:**
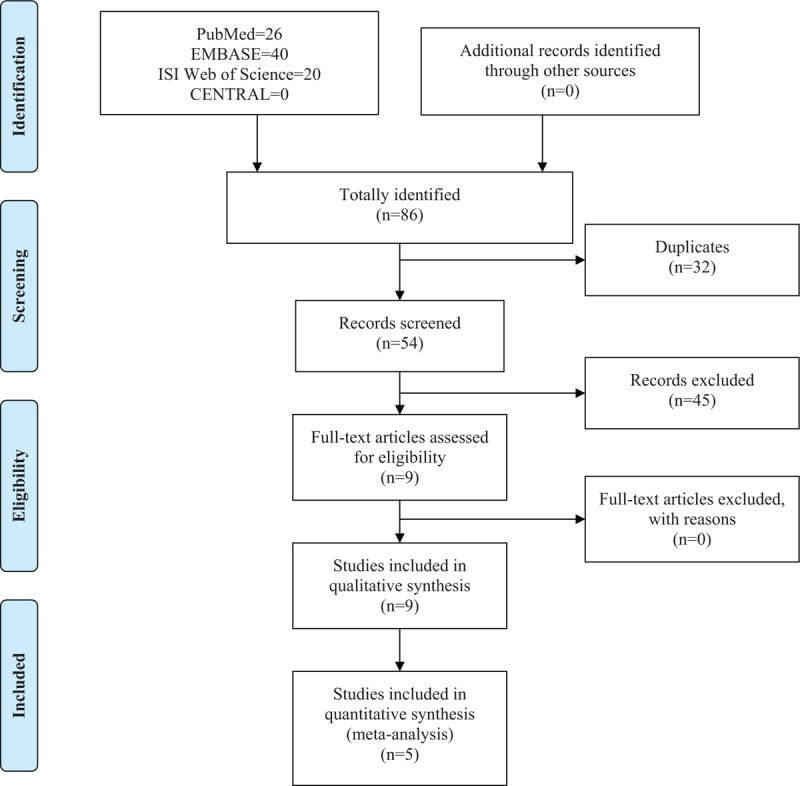
Flow chart of literature search and screen.

The initial search of 4 online databases yielded 86 records (26 from PubMed, 40 from EMBASE, 20 from ISI Web of Science). Any relevant records were not obtained by manual retrieval. After the first scanning, 32 duplicated records were excluded. Of the remaining 54 records, a further 45 citations were eliminated upon screening title and abstract. The rest 9 articles^[22–30]^ were considered eligible for full-text review and went into full-text assessment. None of the articles were removed after assessment by the predefined inclusion and exclusion criteria. Ultimately, 9 studies were incorporated into the qualitative synthesis and 5 studies were further included in the meta-analysis. The literature selection process is presented in Figure 1.

### 3.2 Main characteristics

A summary of main characteristics of the included studies is shown in Table 1.^[22–30]^ Nine case–control studies comprising 6502 CHD patients and 6969 healthy controls were included. All articles were published between 2009 and 2018 in Chinese journals. The sample size of individual research ranged from 380 to 3107. Totally, 9 miRNA genes containing 10 different loci were included in our study: miR-146a (rs2910164), miR-149 (rs2292832), miR-499 (rs3746444), miR-196a2 (rs11614913), miR-218 (rs11134527), miR-34b/c (rs4938723), miR-143 (rs41291957), miR-27a (rs895819), and miR-138 (rs139365823 and rs76987351). Notably, of the 10 loci polymorphisms, rs3746444 in miR-499^[22]^ and rs11614913 in miR-196a2^[25]^ were not compliant with HWE. Besides, Xu et al^[22]^ examined association between several miRNA polymorphisms and CHD risk in 3 stages to reduce the likelihood of false positive findings. None of the studies received <6 stars in NOS for methodological quality assessment, the overall average was 6.5 stars (Table 2).^[22–30]^

**Table 1:**
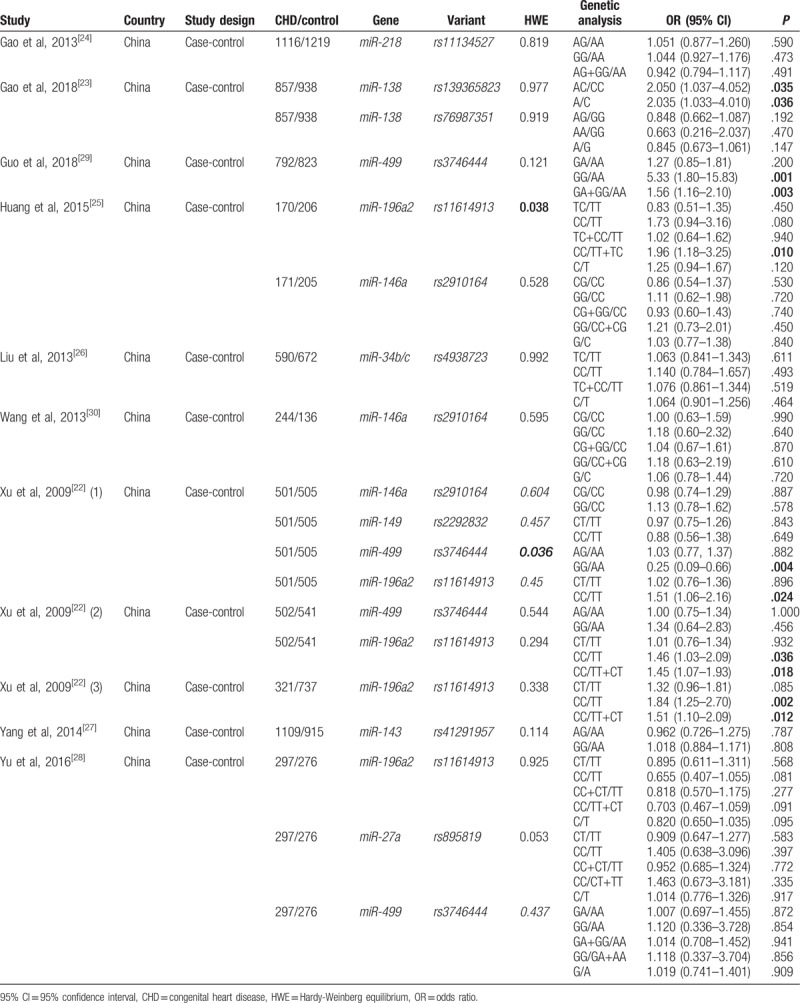
Main characteristics of included studies.

**Table 2:**
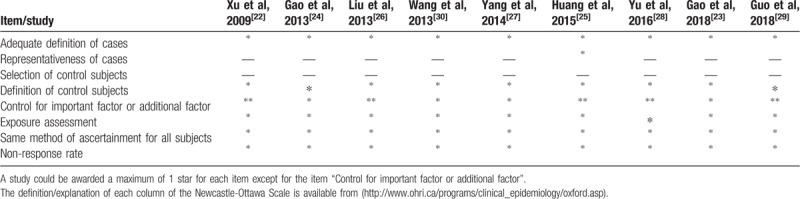
Quality assessment of included studies.

### 3.3 Results of association studies

#### 3.3.1 Strong evidence

Strong evidence was found for the association between rs11614913 polymorphism in miR-196a2 and CHD risk. Rs11614913 polymorphism in miR-196a2 was reported by 5 individual cohorts from 3 studies.^[22,25,28]^ Xu et al^[22]^ found a significant association between rs11614913 and CHD in their 3 cohorts. Genotype CC appeared to significantly increase the CHD risk under homozygous model (CC/TT). The results from each cohort were (OR: 1.51, 95% CI 1.06–2.16; *P* = .024), (OR: 1.46, 95% CI 1.03–2.09; *P* = .036), and (OR: 1.84, 95% CI 1.25–2.70; *P* = .002), respectively. The risk effect of genotype CC under recessive model was detected in stage II (CC/TT+CT, OR: 1.45, 95% CI 1.07–1.93; *P* = .018) and stage III (CC/TT+CT, OR: 1.51, 95% CI 1.10–2.09; *P* = .012).^[22]^ A significant association under the recessive model was observed (CC/TT+TC, OR: 1.96, 95% CI 1.18–3.25; *P* = .01).^[25]^ Nevertheless, these associations were not validated using a relatively small sample size (297 CDH cases and 276 controls).^[28]^

In addition, rs2910164 polymorphism in MiR-146a was examined by three studies.^[22,25,30]^ Association between rs291064 polymorphism in miR-146a and CHD was not determined as strong evidence.

#### 3.3.2 Limited evidence

Limited evidence was found for the association of rs2292832 polymorphism in miR-149 and CHD.^[22]^ Although rs2292832 polymorphism in miR-149 was reported in their stage I cohort with 501 cases and 505 controls, statistical correlation was not found between rs2292832 and CHD risk.

Two loci (rs139365823 and rs76987351) in miR-138 gene were sequenced from 857 CHD patients and 938 healthy controls.^[23]^ Statistical analysis suggested that individuals carrying allele A of rs139365823 had a significant increase of CHD risk by 103.5% (OR 2.035, 95% CI 1.033–4.010; *P* = .036). Carriers of genotype AC had a significantly higher risk (105%) of CHD (OR 2.05; 95% CI 1.037–4.052; *P* = .035). However, a null association was observed between rs76987351 and CHD. Given that only 1 study with low risk of bias reported the association for miR-138 and CHD risk, we determined the level of evidence as limited.

Yu et al^[28]^ reported that rs895819 in miR-27a is not linked with high CHD risk, among 5 genetic models. Furthermore, Gao et al^[24]^ investigated rs11134527 in miR-218 in CHD in a large sample cohort with 1116 cases and 1219 control participants. Statistical analysis did not indicate significant association in both genotype and allele frequencies of rs11134527 polymorphism between CHD cases and the control groups.

Liu e al^[26]^ performed genotyping on 590 CHD patients and 672 healthy controls for rs4938723 polymorphism in miR-34b/c. Significant association was not observed between the distribution of genotype and allelic frequencies.

Moreover, Yang et al^[27]^ evaluated 1109 patients with CHD and 915 controls for rs41291957 polymorphism of miR-143. They were unable to determine significant association of genotype or allele frequencies of rs41291957 with CHD onset.

#### 3.3.3 Conflicting evidence

Conflicting evidence was found for the association of rs3746444 polymorphism in miR-499 and CHD risk. Rs3746444 polymorphism in miR-499 was investigated by 4 individual cohorts from three studies.^[22,28,29]^ Xu et al^[22]^ found that genotype GG acted as a protective factor for CHD risk in stage I cohort (GG/AA, OR: 0.25; 95% CI 0.09–0.66; *P* = .004), but the association was not validated in stage II cohort (GG/AA, OR: 1.34; 95% CI 0.64–2.83; *P* = .456). Similarly, Yu et al^[28]^ did not find significant association between rs3746444 polymorphism in miR-499 and CHD using any genetic model of inheritance. Contrasting Xu et al's findings, Guo et al^[29]^ reported that GG genotype significantly associated with an increased CHD risk (GG vs AA, OR: 5.33; 95% CI 1.80–15.83; *P* = .001).

### 3.4 Meta-analysis results

In addition to the “best-evidence” synthesis method, we used meta-analysis to quantify the association between miRNA polymorphism and CHD risk. Before combining data from each individual study, genetic model of inheritance was not assumed to be made. A model-free approach was used to dictate the best-fitting genetic model for the meta-analysis, to avoid an inflated type I error rate. Three polymorphisms, rs3746444 in miR-499, rs11614913 in miR-192a2, and rs2910164 in miR-146a were included in the meta-analysis, as these were evaluated in no <3 studies and thereby allowing the meta-analysis.

**Figure 2:**
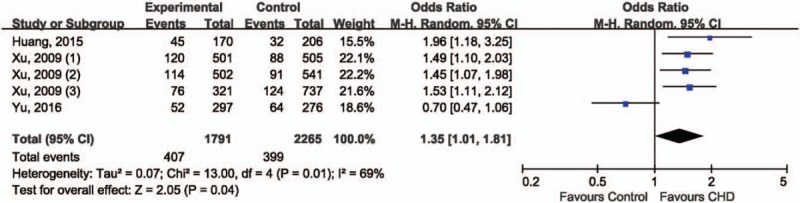
Forest plot of rs11614913 in miR-196a2 and risk of CHD.

**Figure 3:**
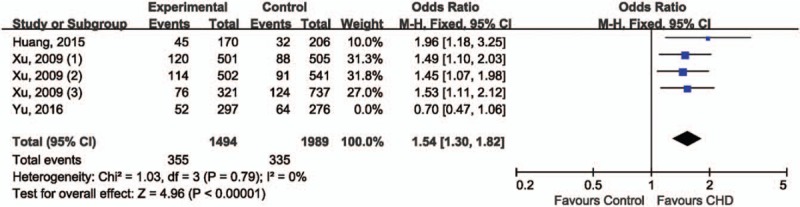
Sensitivity analysis of rs11614913 in miR-196a2 and risk of CHD.

For the association between rs11614913 in miR-196a2 and CHD risk, OR1 (1.40, 95% CI 1.16–1.68; *P* = .0003) and OR3 (1.35, 95% CI 1.14–1.59; *P* = 0.0004) were both statistically significant whereas OR2 (1.04, 95% CI 0.90–1.20; *P* = .60) was not, suggesting that the recessive model (CC vs. CT+TT) could be the best-matching genetic model for meta-analysis. Under recessive model data showed that CC genotype was significantly associated with an increased CHD risk, compared with CT+TT genotype (OR 1.35, 95% CI 1.01–1.81; *P* = .04), the random-effect model was employed for data combination due to statistically significant between-study heterogeneity (*I*^2^ = 69%, *P* = .01) (Fig. 2). The reliability of combined results was further confirmed by the leave-one-out sensitivity analysis. Direction of association between rs11614913 and CHD risk remained unchanged after removing included study. Interestingly, the between-study heterogeneity became insignificant after the exclusion of Yu et al's study, suggesting that that study could be the potential source of inter-study variability. Thus, this study was removed from the meta-analysis. Data from the other 4 studies indicated that CC genotype of rs11614913 was significantly associated with an increased CHD risk comparing with CT+TT genotype (OR 1.54, 95% CI 1.30–1.82; *P* < .00001) (Fig. 3). Funnel plot was not generated due to limited number of included studies. The Egger test (*t* = −0.01, *P* = .995) and Begg test (*z* = 0.73, *P* = .462) suggested absence of statistically significant publication bias.

**Figure 4:**
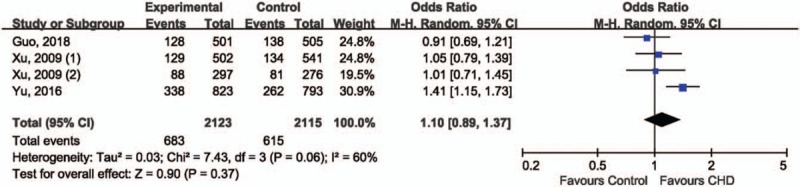
Forest plot of rs3746444 in miRNA-499 and risk of CHD.

For the association between rs3746444 in miRNA-499 and CHD risk, the recessive model was deemed as the most plausible genetic model for meta-analysis because OR1 (1.64, 95% CI 1.16, 2.32; *P* = .006) and OR3 (1.46, 95% CI 1.02–2.09; *P* = .04) were statistically significant, whereas OR2 (1.11, 95% CI 0.97–1.27; *P* = .14) was not. The pooled data suggested no association between GG genotype of rs3746444 and risk of CHD comparing with GA+AA genotype (OR 1.10, 95% CI 0.89–1.37; *P* = .37), in random effect model (*I*^2^ = 60%, *P* = .06) (Fig. 4). The robustness and reliability of pooled results were confirmed by sensitivity analysis (data not shown). The Egger test (*t* = −2.17, *P* = .163) and Begg test (*z* = 0.34, *P* = .734) suggested absence of statistically significant publication bias.

To determine the association between rs2910164 in miR-146a and CHD risk, we utilized a model-free approach. Association between rs2910164 in miR-146a and CHD (OR1: 1.11, 95% CI 0.84–1.47; OR2: 0.98, 95% CI 0.79–1.21; OR3: 1.15, 95% CI 0.88–1.50) was not observed, consistent with “best-evidence” synthesis results.

## 4 Discussion

We summarized available literature from 9 studies^[22–30]^ and presented analysis on the associations of reported genetic polymorphisms in several miRNA genes and CHD risk susceptibility. Included studies suffered from considerable heterogeneity as very few studies shared the same loci. Therefore, we assessed each study and locus polymorphism individually and evaluated whether certain polymorphism may associate with CHD using “best-evidence” synthesis method. For a better understanding of the association between miRNA polymorphism and CHD risk, a model-free approach was employed to quantify the associations. Only rs11614913, rs3746444, and rs2910164 polymorphisms were included in the meta-analysis as sufficient number of studies examined these three polymorphisms. Among these included SNPs, strong evidence suggested that rs11614913 in miR-196a2 showed significant association with CHD risk, which was further supported by meta-analysis, using recessive model. However, rs2910164 in miR-16a and rs3746444 in miR-499 were not associated with altered CHD risk, as suggested by both “best-evidence” synthesis and meta-analysis. Limited evidence was found to support an association between other polymorphisms and CHD susceptibility.

The exact etiology of CHD is not defined yet. Furthermore, intrinsic factors, in collaboration with external factors, demonstrated to drive the occurrence and development of CHD. Genetic factors, as intrinsic factors, play an important role during this process.^[39,40]^ MiRNAs have wide-ranging effects on mRNA transcripts,^[41]^ with improved recognition of various genetic variants associated with different diseases. Polymorphisms in miRNA genes have received great attention over the past decade. Although occurrence of polymorphisms in miRNA sequences is relatively rare, it is of great importance.^[42]^ MiRNA SNPs were linked to a several complicated diseases, such as diabetes mellitus,^[43]^ hypertension,^[44]^ malignant tumors,^[45–47]^ stroke,^[48]^ and several other diseases.^[49]^

Furthermore, miRNAs are demonstrated to be involved in cardiovascular development and heart diseases. Dysregulation of miRNAs has been reported to cause structural abnormalities in the vasculature and heart.^[50]^ Rs2910164 in miRNA-146a has a C>G substitution leading to mispairing in the 3’ arm of the miR-146a and thereby affecting the third base in the seed region of has-miR-146a-3p.^[51]^ However, changes induced by either alleles of this polymorphism have not been established.^[51]^ Furthermore, C allele of rs2910164 was correlated with enhanced expression of miRNA compared with G allele. However, G allele was shown to upregulate miR-146a expression in complex diseases, ranging from type 2 diabetes mellitus to thyroid carcinoma.^[52–54]^ Based upon these discrepancies, we speculate that disease-specific, cell type-specific or tissue type-specific factors alter the effect of rs2910164 on miR-146a function. Here, rs2910164 polymorphism in miR-146a did not alter the CHD risk, evidenced by both “best-evidence” synthesis and model-free meta-analysis. This polymorphism might change expression of miR-146a outside the heart tissue and not have pronounced effect on structural abnormalities, as the expression of miR-146a is primarily found in the vascular smooth muscle cells. However, potential effects of miR-146a on the heart development and structural abnormalities need to be investigated.

MiR-196a was identified as an upstream regulator of Hoxb8 and Sonic hedgehog (Shh), which are required throughout, from cardiac septation to valve formation.^[55,56]^ MiRNAs bind to complementary sequences within the mRNAs of their target genes and inhibit their expression by either translational repression or mRNA degradation. CC of rs11614913 in miR-196a2 was significantly associated with increased expression of mature miR196a in cardiac tissue specimens of patients with CHD.^[22]^ As a result, the increased expression of miR-196a suppressed mRNA target of Hoxb8. Thus, miR-196a-Hoxb8-Shh signaling pathway may play an important role in heart development and possibly get involved in several subtypes of CHD, through errors in cardiac septation, outflow tract morphogenesis and valve formation.^[22]^ Four of 5 independent cohorts reported a strong association between rs11644913 and CHD risk. These findings were further supported by meta-analysis of 5 individual datasets and subsequent sensitivity analysis. Notably, except for Yu et al's study, all other 4 independent comparisons indicated that CC was significantly associated with an increased CHD risk compared with TC+CC genotype. In Yu et al's study, the miR-196a2 homozygous CC variant and C allele were negatively associated with atrial septal defect, but not with the pooled CHD risk. If the pathogenesis varies between different CHD subtypes, the conclusion drawn from the pooled CHD groups might be contradictory to that from a specific CHD subtype. However, in our current meta-analysis, the overall size effect remained statistically significant with or without the inclusion of Yu et al's study, suggesting an overall detrimental effect of CC variant on CHD. The future studies should focus on the potential effect of miR-196a on different CHD subtypes.

MiR-499 is primarily expressed in the cardiac cells and skeletal muscles. During the process of cardiomyocyte differentiation, expression of miR-499 is dynamically regulated.^[57]^ The polymorphism included by our study, rs3746444 in miR-499, resides in the seed region of miR-499a-3p and the 3’ region of miR-499b-5p. Rs3746444 can either alter the miR-499a-5p/3p expression or affect the repertoire of target genes of miR-499a-3p or even a combination of both conditions. A higher frequency of a certain SNP allele or genotype in a series of individuals affected with a disease could be interpreted as meaning that the tested variant increases the risk of a specific disease. In contrast to the stage I study from Xu et al,^[22]^ where they found a significantly higher frequency of GG genotype in miR-499 rs3746444 than AA (P = .004), our current meta-analysis failed to demonstrate a significant association between rs3746444 and CHD. “Best evidence” synthesis also yielded conflicting results. However, in the stage II study of Xu et al, when the control subjects conformed to HWE (*P* = .544), the researchers failed to replicate the significant finding. Departure from HWE in control subjects may be caused by genotyping error, assortative matching, population stratification and chance. Nonexclusively, departure from HWE in the control samples was also detected in Huang et al's study.^[25]^ It still remains unclear how rs3746444 gets involved in the pathogenesis of CHD, further research is needed to clarify the effect of this polymorphism induced changes in CHD development.

In addition to the mechanisms discussed above, other polymorphisms of miRNAs are involved in cardiovascular development and heart diseases. He et al demonstrated that upregulation of miR-138 had a protective effect of myocardial adaptation to chronic hypoxia by inhibiting hypoxia-induced apoptosis.^[58]^ Further, He et al observed that overexpression of miR-23b might promote the apoptosis of cardiomyocytes and decrease cell growth in a hypoxic condition.^[59]^ Morton et al reported that miR-138 could help establish distinct domains of gene expression during cardiac morphogenesis, and disruption of miR-138 could result in abnormal cardiac morphology by targeting the retinoic acid pathway in zebrafish.^[60]^ Moreover, Nishi et al demonstrated that miR-27a regulated beta cardiac myosin heavy chain gene expression by targeting thyroid hormone receptor beta in cardiomyocytes.^[61]^ A research by Chiavacci et al suggested that miR-218 regulates the expression of a gene encoding Tbx5a, which is one of the key transcription factors mediating heart development in vertebrates.^[62]^ Downregulation of miR-218 was able to rescue the heart defects caused by over-expression of Tbx5.^[62]^ Fish et al^[63]^ demonstrated that miR-218 was involved in the process of heart tube formation. The study by Deacon et al^[64]^ revealed that the miR-143-adducin3 genetic pathway were essential for cardiac chamber formation and through adjusting myocardial cell morphology. All these studies suggested the importance of miRNAs to heart development, but due to limited number of studies included more studies are encouraged to elucidate the potential effect of these variants on CHD.

Another noted phenomenon noted in our included studies was that all the included studies applied multiple genetic models of inheritance to evaluate the association between miRNA polymorphisms and CHD risk, but without an appropriate correction for multiple tests. Type I error (false-positive results) may arise through a failure to correct for multiple testing, and including correction for multiple testing will definitely increase the sample sizes required. Therefore, studies for which association *P* values were not far below predetermined threshold should be questioned and checked for genotyping quality. Our inability to draw a definite conclusion of association between miRNA polymorphisms and CHD risk also indicated that further studies with larger sample-size, better genotyping quality, and within different cultural background are needed.

Compared to other said studies, our study presented a summary of various CHD-related miRNA polymorphisms with their ORs, 95% CI, and *P* values, regardless of positive or negative association. Meta-analyses were also performed using a model-free approach to quantify the associations, which allowed to choosing the SNP or miRNA for future investigations. Several limitations were pointed out in this systematic review. First, all the included studies were case–control designed and hospital population-based, leading to potential selection bias of included participants and a low evidence level in the present study. Second, only Chinese population were included in our study. Some polymorphisms are ethnicity-specific, and thus present either stronger, weaker, or even no effect on a different race. Therefore, our results could not be extrapolated to wide-ranging populations. Third, 9 articles from four databases were retrieved for the analysis. Relevant articles published in other languages might have been skipped. Finally, identifying genetic factors responsible for CHD was on demand. Generally, identification of a novel locus or gene leads to loss of interest among researchers to study it further. Despite several polymorphisms in diverse miRNA genes described in previous studies, very few were investigated by other researchers. Those studies, therefore, only allowed a systematic review but were not appropriate for meta-analysis.

## 5 Conclusion

Taken together, locus polymorphisms in miRNAs are not generally associated with CHD. We found some genetic variants that potentially contribute to the CHD risk. Strong evidence suggested an association between rs11614913 in miR-196a2 and CHD risk, which was further confirmed by meta-analysis involving 5 independent comparisons. Strong evidence indicated lack of association between rs2910164 in miR-146a and CHD. Limited or conflicting evidence were found in the existing literature for the correlations between other miRNA polymorphisms and CHD risk. Further studies are necessary involving larger populations of different ethnicities to obtain a better understanding of these associations.

## Author contributions

**Conceptualization:** Zheng-tao Lv.

**Data curation:** Xing-yan Li, Kun Chen, Zheng-tao Lv.

**Formal analysis:** Xing-yan Li, Kun Chen, Zheng-tao Lv.

**Investigation:** Xing-yan Li, Kun Chen.

**Methodology:** Xing-yan Li, Kun Chen.

**Resources:** Xing-yan Li, Kun Chen, Zheng-tao Lv.

**Software:** Xing-yan Li, Kun Chen, Zheng-tao Lv.

**Supervision:** Zheng-tao Lv.

**Validation:** Xing-yan Li, Kun Chen, Zheng-tao Lv.

**Visualization:** Xing-yan Li, Kun Chen, Zheng-tao Lv.

**Writing – original draft:** Xing-yan Li, Kun Chen.

**Writing - review & editing:** Zheng-tao Lv.

Zheng-tao Lv orcid: 0000-0002-8238-7194.
